# Optical Anisotropy and Excitons in MoS_2_ Interfaces for Sensitive Surface Plasmon Resonance Biosensors

**DOI:** 10.3390/bios12080582

**Published:** 2022-07-29

**Authors:** Amir Eghbali, Andrey A. Vyshnevyy, Aleksey V. Arsenin, Valentyn S. Volkov

**Affiliations:** Center for Photonics and 2D Materials, Moscow Institute of Physics and Technology (MIPT), 141700 Dolgoprudny, Russia; eghbali.amir@phystech.edu (A.E.); arsenin.av@mipt.ru (A.V.A.); volkov.vs@mipt.ru (V.S.V.)

**Keywords:** surface plasmon resonance, transition metal dichalcogenides, MoS_2_, sensitivity enhancement

## Abstract

The use of ultra-thin spacer layers above metal has become a popular approach to the enhancement of optical sensitivity and immobilization efficiency of label-free SPR sensors. At the same time, the giant optical anisotropy inherent to transition metal dichalcogenides may significantly affect characteristics of the studied sensors. Here, we present a systematic study of the optical sensitivity of an SPR biosensor platform with auxiliary layers of MoS_2_. By performing the analysis in a broad spectral range, we reveal the effect of exciton-driven dielectric response of MoS_2_ and its anisotropy on the sensitivity characteristics. The excitons are responsible for the decrease in the optimal thickness of MoS_2_. Furthermore, despite the anisotropy being at record height, it affects the sensitivity only slightly, although the effect becomes stronger in the near-infrared spectral range, where it may lead to considerable change in the optimal design of the biosensor.

## 1. Introduction

Biosensors based on surface plasmon resonance (SPR) are widely recognized for their high sensitivity, stability, and fabrication simplicity [1,2]. Since the initial proof of principle four decades ago [3,4], SPR biosensors have been significantly improved and are now commercially available. Nevertheless, the ability of SPR biosensors to detect small molecules remains limited, and further improvements are required.

(1) $$\frac{\Delta P}{\Delta C}=\frac{\Delta P}{\Delta n}\frac{\Delta n}{\Delta C}=S_{\mathrm{RI}}E.$$

The sensitivity of a biosensor, defined as the ratio of the change in the output signal Δ*P* to the change in the analyte concentration Δ*C*, is determined by the physical sensitivity to the refractive index change *S*_RI_ and the immobilization efficiency *E*, which quantifies the refractive index change caused by Δ*C*:

As a result, the development of biosensors involves both the improvement of the optical sensitivity [5,6] and the search for better sensing surfaces [7,8,9]. The successful isolation of graphene in 2004 [10] followed by the discovery of a broad family of van der Waals (vdW) materials [11,12] introduced a new degree of freedom for the design of biosensors, since all such materials can be easily transferred on any substrate and combined together to form vdW heterostructures. 

The study of sensitivity enhancement with vdW materials has become a hot topic in the last decade, since they are promising both as immobilization layers [13,14,15,16,17] and as optical sensitivity enhancing layers [16,17,18,19,20,21,22,23,24,25,26,27,28]. Furthermore, additional layers of graphene or other materials can protect the metal from the environment, thereby improving the stability of the sensor structure [29]. 

The optical response of the majority of semiconductor vdW materials is dominated by excitons, pairs of electrons and holes bound by the Coulomb interaction. Owing to the reduced Coulomb interaction screening in 2D, exciton-binding energies reach up to 500 meV, which allows them to persist at room temperature. As a result, such materials possess high refractive indices and strongly absorb light at excitonic resonances [30]. Furthermore, vdW materials possess the structural anisotropy that entails the strong anisotropy of optical properties [31,32,33]. Furthermore, certain vdW materials, such as MoS_2_, exhibit nontrivial transformation of their band structure when thinned down to a single atomic layer, leading to the formation of a direct gap in an originally indirect-gap semiconductor [34]. However, in literature on the application of vdW materials for biosensing, the isotropic optical constants are typically used. This fact casts doubts on the achieved results, since SPR resonance is observed for p-polarized light, which is sensitive to the out-of-plane dielectric constant of the vdW material. Furthermore, the analysis of the biosensor performance is usually restricted to a wavelength of 633 nm, which is justified by the properties of the available measurement setups, but such an approach does not allow one to gain insight into the role of excitons in sensitivity enhancement. Finally, with the present maturity of laser technology, the wavelength of the sensing beam is not limited to 633 nm. 

In this work, we report a broadband study of the SPR biosensor platform performance enhancement via auxiliary MoS_2_ layers with the focus on influence of excitons and optical anisotropy. We consider the angle and phase interrogation measurement schemes in the Kretschmann-type configuration. We find a moderate effect of anisotropy and explain it by calculating effective refractive indices of the anisotropic layer. Excitonic resonances lead to dips in the maximum angular sensitivity and the optimal thickness of the MoS_2_ layer at which it is reached. At the same time, the sensitivity in the off-resonant spectral range strongly benefits from the high refractive index and low optical absorption of MoS_2_. For the phase interrogation scheme, we find that the phase sensitivity is determined by how closely the biosensor operates to a zero-reflection point. The closer the zero-reflection point, the higher the sensitivity. The anisotropy and excitonic resonances only shift the position of zero-reflection points but do not make them disappear. We conclude that, for the best performance, it is highly desirable to have the ability to tune the operation wavelength.

## 2. Methods

**Figure 1:**
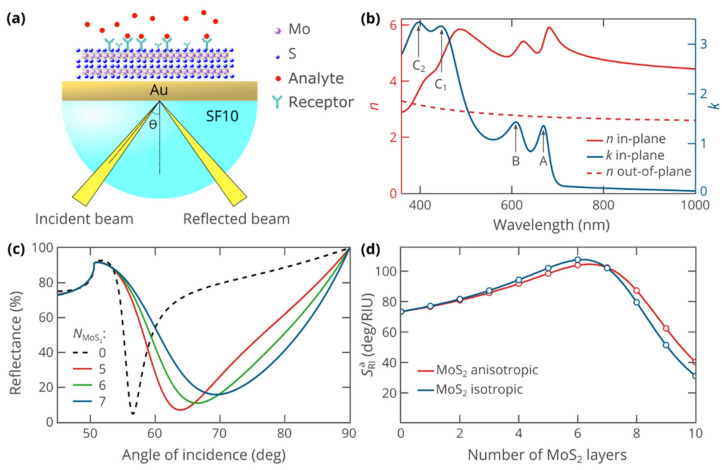
(**a**) Schematic view of the studied SPR biosensor operation; (**b**) Anisotropic optical properties of MoS_2_ nanosheets [32,36]. “A, B, C_1_, C_2_” label the excitonic absorption peaks; (**c**) Reflectance as a function of the angle of incidence at a wavelength of 633 nm and varying number $N_{{\mathrm{MoS}}_{2}}$ of MoS_2_ layers. Black dashed curve represents the reference biosensor without the MoS_2_ cover ($N_{{\mathrm{MoS}}_{2}}=0$ ). SPR curves are plotted assuming that the analyte layer is pure water; (**d**) The sensitivity of SPR biosensor as a function of the thickness of the MoS_2_ layer. In calculations using isotropic optical properties of MoS_2_, we set $\varepsilon =\varepsilon_{∥}$.

For calculations of the sensitivity of biosensors, a complex amplitude of reflection for p-polarized light *r_p_* is required. To calculate it, we employed the transfer matrix method (TMM) for multilayered structures composed of materials with arbitrary dielectric permittivity tensors [35] implemented in open-source PyLlama library for Python programming language. The biosensor structure comprised (Figure 1a): (i) SF10 glass optical prism; (ii) 40-nm-thick gold layer; (iii) variable number of atomic layers of MoS_2_; (iv) sensing medium. In simulations, we used Yakubovsky et al. data for thickness-dependent optical properties of the gold layer [36] and recently obtained results on the optical constants of MoS_2_ and their anisotropy (Figure 1b) [32,37]. The optical axis was set normally to the gold surface. The unperturbed refractive index of the sensing medium was equal to that of water [38]. The optical constants of SF10 glass were taken from the manufacturer’s datasheet [39].

After the calculation of reflectance $R_{p}={\left|r_{p}\right|}^{2}$ as a function of the incidence angle *θ*, we determined the sensitivity as the ratio of shift in the reflectance minimum Δ*θ* to the change in the refractive index Δ*n* of the sensing medium:

(2) $$S_{\mathrm{RI}}=\frac{\Delta \theta }{\Delta n}.$$

In calculations of sensitivity for the phase interrogation measurement scheme, we evaluated reflection amplitudes for both s and p polarizations of incoming light *r_s_* and *r_p_*, correspondingly, and then obtained a differential phase:

(3) $$\varphi_{d}=\arg\left(r_{p}/r_{s}\right),$$

Eventually, the sensitivity was obtained:

(4) $$S_{\mathrm{RI}}=\left|\frac{\Delta \varphi_{d}}{\Delta n}\right|.$$

## 3. Results

### 3.1. Angle-Interrogation Scheme

We consider a common Kretschmann-type SPR sensor platform depicted in Figure 1a. In our design, the layer of gold is not in direct contact with the analyte, separated from it by several atomic layers of MoS_2_. The use of a highly refractive SF10 prism allows us to achieve the optimal sensitivity with a higher number of MoS_2_ layers, which slightly enhances the influence of excitons and anisotropy and makes it easier to analyze. Note that the MoS_2_ layer can be directly covered by receptors thanks to the high affinity of van der Waals materials with organic molecules [40] or an additional layer of graphene oxide, whose superior immobilization properties have been well established. Figure 1b shows the optical properties of MoS_2_, indicating its relevant features, namely, giant optical anisotropy and A, B, C_1_, and C_2_ excitons, responsible for its strong absorption and very high refractive index in the visible and near-infrared range (please note that the double absorption peak of bulk MoS_2_ around 400 nm overlaps with the C-exciton of monolayer MoS_2_ [37], hence the labels C_1_ and C_2_). Absence of excitonic features in the out-of-plane component of refractive index is due to the in-plane dipole moment of intralayer optically active excitons in MoS_2_ [32,41,42]

SPR resonance was simulated by the transfer matrix method for anisotropic materials (see Methods). The reflectance of p-polarized waves shows the characteristic dip at an angle of incidence, at which the in-plane component of the incident wavevector matches the wavenumber of surface plasmon polaritons (Figure 1c). Upon increase in the number of atomic layers of MoS_2_, the resonance dip shifts to higher angles of incidence owing to the increased wavenumber of the guided SPP wave. At the same time, the resonance becomes wider, which agrees with the previous studies of SPR in systems with additional dielectric layers [5,18,43]. While the increase in FWHM of the resonance makes it harder to precisely determine the location of SPR dip, the use of advanced data processing techniques allows one to overcome FWHM limitations to a certain extent (more details in Reference [18]). More importantly, the angular sensitivity *S*_RI_ reaches its maximum when the gold layer is covered by six-layered MoS_2_ film (Figure 1d). The maximum sensitivity of 105 deg/RIU exceeds the value for the bare gold film by 42%.

To investigate the impact of the giant optical anisotropy of MoS_2_, we have additionally performed similar sensitivity calculations, assuming the optical response of MoS_2_ to be fully isotropic. Interestingly, in spite of the record-high birefringence, the sensitivity curves in Figure 1d only moderately deviate, although the deviation increases with the increase in the number of MoS_2_ layers. They predict the maximum sensitivity at the same MoS_2_ thickness of six atomic layers, while the maximum sensitivity in the isotropic case is by 3.2% higher than in the anisotropic one. At the same time, we note that the sensitivity is only partly determined by the properties of the MoS_2_ layer and the influence of the anisotropy on that MoS_2_-induced sensitivity enhancement $\Delta S_{\mathrm{RI}}^{a}$ is as high as 11% for the six-layer film.

**Figure 2:**
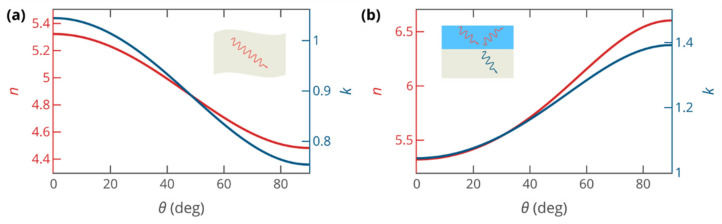
Effective refractive indices of the anisotropic MoS_2_ layer, governing: (**a**) phase accumulation and field attenuation upon propagation of a p-polarized wave (*n*_eff_ = *β*/*β*_0_) and (**b**) reflection and transmission of a p-polarized wave at interfaces with other materials, calculated from Equation (5).

To understand why the sensitivity depends on the out-of-plane refractive index so weakly, we calculated the properties of the isotropic layer equivalent to the anisotropic MoS_2_ layer. The properties of the anisotropic layer can be homogenized using two different approaches. In one of them, we determined the effective refractive index as $n_{\mathrm{eff}}^{p}=\beta /\beta_{0}$, where *β* is the wavenumber of the plane wave, propagating through the medium, and *β*_0_ = *ω*/*c* is the wavenumber in vacuum. As a result, the wavevector in the effective isotropic medium is the same as in the anisotropic one. Within the second approach, we required that the amplitudes of the reflected and the transmitted waves through the interface between the anisotropic material and other media remain the same upon replacing the anisotropic medium with an effective isotropic medium. In the case of p polarization, relevant to SPR, this leads to the following equation for $\varepsilon_{\mathrm{eff}}^{i}={\left(n_{\mathrm{eff}}^{i}\right)}^{2}$ (see Appendix A for the derivation):

(5) $$\frac{\beta_{0}^{2}}{\varepsilon_{∥}}-\frac{\beta_{∥}^{2}}{\varepsilon_{⊥}\varepsilon_{∥}}=\frac{\beta_{0}^{2}}{\varepsilon_{\mathrm{eff}}^{i}}-\frac{\beta_{∥}^{2}}{{\left(\varepsilon_{\mathrm{eff}}^{i}\right)}^{2}},$$

where the in-plane component of the incident beam wavevector is $\beta_{∥}=\beta_{0}n_{\mathrm{SF}10}\sin\theta$, with *θ* being the angle of incidence. The effective index obtained using the first approach is responsible for phase accumulation and attenuation caused by propagation through the anisotropic layer, while the index given by the second approach governs scattering on the interfaces between the anisotropic layer and other media. Evidently, for s-polarized waves, both approaches yield the same effective index value $n=\sqrt{\varepsilon_{∥}}$, that is, the ordinary refractive index. By contrast, the effective indices of MoS_2_ for p-polarized waves, given by these approaches, are different and depend on the incidence angle *θ*, as shown in Figure 2a,b.

At an angle *θ* = 66° corresponding to the minimum reflectance, the effective refractive indices $n_{\mathrm{eff}}^{p}$ = 4.63 + 0.81*i* and $n_{\mathrm{eff}}^{i}$ = 6.29 + 1.32*i* differ from the in-plane refractive index by about 15%, a striking contrast with a difference of almost a factor of 2 between in-plane and out-of-plane refractive indices of MoS_2_. Our calculation not only explains the limited effect of optical anisotropy on sensitivity enhancement but elucidates the origin of the challenges arising in the measurement of anisotropic optical constants of high-refractive-index materials. In the case of thin-film ellipsometry, the incident beam propagates in air, which limits the relative difference between the effective optical constants and the in-plane refractive index to below 5%, thereby making ellipsometric measurement of anisotropy very hard. Thus, to measure out-of-plane optical properties, one should employ more complicated techniques that involve probing of the planar waveguide modes by near-field optical microscopy [32] or growing thick monocrystalline samples of the studied materials [33].

**Figure 3:**
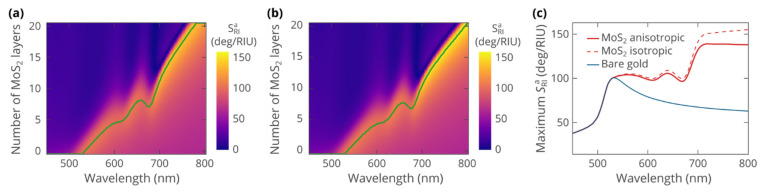
Heatmaps of the angular sensitivity of the SPR biosensor as a function of the operating wavelength and the number of MoS_2_ atomic layers covering gold. The heatmaps were calculated assuming: (**a**) anisotropic optical properties of MoS_2_; (**b**) isotropic dielectric function $\varepsilon =\varepsilon_{∥}$ of MoS_2_. Solid green line shows the dependence of the optimal thickness on the wavelength. (**c**) Maximum angular sensitivity of the biosensor versus the operating wavelength. The sensitivity of the sensor without MoS_2_ cover (blue curve) is added for reference.

To reveal the excitonic effects, we have evaluated the optical sensitivity of the biosensor as a function of the operating wavelength and the number of MoS_2_ layers. Figure 3a,b indicates that SPR sensing in the studied system is possible at wavelengths above 500 nm. Starting from 540 nm, the use of MoS_2_ enhances the sensitivity of the biosensor. Furthermore, the optimal thickness correlates with the excitonic peaks of MoS_2_ showing two distinct features related to A and B excitons. Particularly, due to excitonic resonances, the optimal thickness, required to achieve maximum sensitivity, decreases. Moreover, the maximum achievable sensitivity shows dips at peaks of optical absorption. At the same time, at wavelengths above the excitonic absorption tails, the biosensor strongly benefits from the ultra-high refractive index of MoS_2_, originating from excitons via the Kramers–Kronig relations. Above 700 nm, the angular sensitivity reaches 140 deg/RIU, which is about twice the sensitivity of the bare gold sensor at the same wavelengths (Figure 3c).

Comparison between the heatmaps calculated with anisotropic (Figure 3a) and isotropic (Figure 3b) optical properties of MoS_2_ confirms the relatively weak influence of anisotropy. Heatmaps show the same features and behavior, although the value of angular sensitivity is slightly lower in the anisotropic case. At the same time, we notice that the effect of anisotropy is more pronounced when more MoS_2_ layers are involved. For instance, the optimal thickness of 17 layers obtained from anisotropic calculations at 745 nm is by 2 higher than the value produced, assuming isotropic optical response of MoS_2_. This implies that for thicker layers of MoS_2_, at wavelengths above 700 nm, the anisotropy should not be neglected.

### 3.2. Phase Interrogation Scheme

While the use of additional layers produces the possibility of improving the angular sensitivity, the sensitivity enhancement remains limited. To overcome these limitations, Kabashin and Nikitin proposed to measure the phase of the reflected signal rather than its intensity [44,45]. This scheme leverages the strong variation in the phase in the vicinity of the reflectance minimum (Figure 3a) to achieve enhancement of the measured signal and its sensitivity to the refractive index of analyte solution.

**Figure 4:**
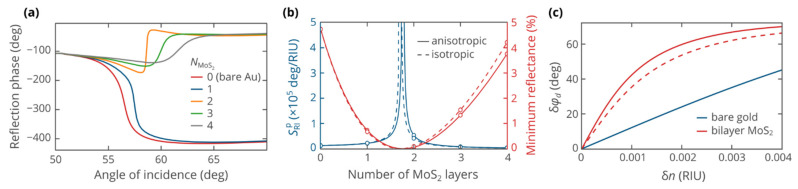
(**a**) The phase of the reflected p-polarized wave as a function of the angle of incidence, plotted at a variable number of atomic layers of MoS_2_. (**b**) The phase sensitivity and the minimum reflectance versus the thickness of MoS_2_ cover, showing the singularity of sensitivity at a zero-reflection point. (**c**) Dependence of the biosensor signal on the refractive index change. The operating wavelength in all panels was set to 633 nm.

If the thickness changes continuously, the phase sensitivity grows infinite upon approaching the zero-reflection thickness (Figure 4b), owing to the phase singularity of zero. Our result complements the previous works reporting orders of magnitude of increase in the phase sensitivity upon deposition of ultra-thin layers of materials [46,47]. Importantly, the singularity in the sensitivity is present in cases of both isotropic and anisotropic dielectric tensors for the MoS_2_ layer, which we attribute to the topological protection of zero-reflection points, demonstrated recently [48]. However, there are a few obstacles that limit the applicability and maximum sensitivity of phase-interrogation of SPR biosensors. First, as one gets closer to a zero-reflection point, the range of measurable refractive index changes shrinks (Figure 4c), with a maximum detectable refractive index change of the order of $\delta n_{\max}\sim 180{}^{\circ}/S_{\mathrm{RI}}^{p}$. More importantly, as evident from the name “zero reflection”, the measurement of phase is challenging due to the vanishingly small intensity of the reflected light. Near a zero-reflection point, the measured phase may be strongly affected by the noise and nonmonochromaticity of the incident beam. This led to doubts on the feasibility of the measurement procedure [49], which, nevertheless, can be overcome by an ellipsometric scheme of differential phase measurement [50,51]. Finally, the thickness of the deposited MoS_2_ film is not continuous but rather a discrete value determined by the integer number of atomic layers.

**Figure 5:**
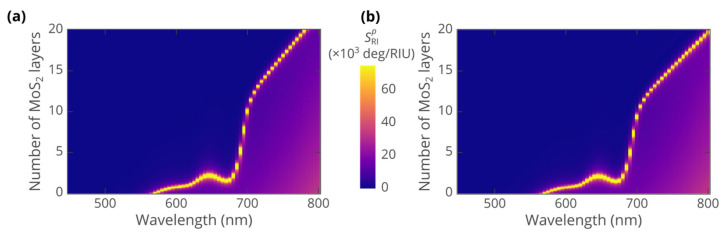
Heatmaps of phase sensitivity as functions of the number of atomic layers in MoS_2_ film and the operating wavelength. Calculations were performed using: (**a**) full anisotropic dielectric tensor and (**b**) isotropic dielectric permittivity for the MoS_2_ layer.

Some of the above issues can be overcome, at least partly. The wavelength-dependent study of the phase sensitivity shows that zero-reflection points form a continuous line in the wavelength-thickness domain (Figure 5a,b). Therefore, by tuning the wavelength, a zero-reflection point can be accessed in a realistic setup. For instance, zero reflection can be achieved with bilayer MoS_2_ at a wavelength of 655 nm. Furthermore, in a real experimental setup, the optical properties of MoS_2_ are affected by the environment, deposition and transfer techniques, and structural properties of produced film. These factors shift the parameters necessary for zero reflection from the calculated values, which again necessitates the wavelength tuning. Finally, by tuning the operating wavelength, one is able to control the distance from the zero-reflection point, thereby setting the operation regime that is optimal for the available detection equipment. Furthermore, one may adjust the operational range of the biosensor by getting closer to or farther from the zero-reflection point.

## 4. Conclusions

To summarize, we theoretically studied the performance of the SPR biosensor platform with an auxiliary MoS_2_ layer in a broad spectral range, accounting for the giant optical anisotropy of MoS_2_. In spite of a difference of almost a factor of 2 between out-of-plane and in-plane refractive indices, the impact of the anisotropy on the sensitivity turned out to be weaker than expected. This can be explained by a modest 15% difference between the effective indices of the anisotropic layer and the in-plane refractive index of MoS_2_. The analysis of the excitonic influence shows contrasting behavior in the resonant and off-resonant spectral range. Close to the excitonic resonances, the optimal thickness of MoS_2_ and maximum angular sensitivity exhibit distinct dips, while the off-resonant operation of the sensor benefits from the high refractive index and low optical absorption. The maximum sensitivity with the MoS_2_ layer exceeds the sensitivity of the biosensor without the MoS_2_ cover by almost a factor of 2.

The phase interrogation scheme is found to be less affected by anisotropy and exciton-driven optical response of MoS_2_, because the phase sensitivity is determined by how close to a zero-reflection point the sensor operates and becomes formally infinite at that point. We find that these points form a line in the wavelength-MoS_2_ thickness plane. Therefore, it is possible to get very close to such a point if there is a possibility of tuning the operation wavelength. The zero-reflection line slightly shifts upon the turn-off of anisotropy. We connect the robustness of the zero-reflection line to the changes in optical properties and the topological protection of zero-reflection points. This protection also ensures the availability of zero-reflection points even if the optical properties of the MoS_2_ layer are different from the values used in calculations, due to structural or environmental influence.

Our results draw a broader picture of the use of van der Waals materials for the enhancement of biosensors, thereby providing a firm ground for future technological developments.

## Data Availability

Not applicable.

## Appendix A

In this Appendix A, we derive closed-form expressions for effective refractive indices of a planar anisotropic layer with a dielectric tensor $\varepsilon =\mathrm{diag}(\varepsilon_{∥},\varepsilon_{∥},\varepsilon_{⊥})$ for p-polarized waves.

The effective index related to wave propagation was defined as $n_{eff}^{p}=\beta /\beta_{0}$, where *β* is the wavenumber of the plane wave, propagating through the medium, and *β*_0_ = *ω*/*c* is the wavenumber in vacuum. Starting from the dispersion law for p-polarized waves

(A1) $$\frac{\beta_{∥}^{2}}{\varepsilon_{⊥}}+\frac{\beta_{⊥}^{2}}{\varepsilon_{∥}}=\beta_{0}^{2},$$

we inferred:

(A2) $$\beta =\sqrt{\beta_{∥}^{2}+\beta_{⊥}^{2}}=\sqrt{\beta_{∥}^{2}+\varepsilon_{∥}\left(\beta_{⊥}^{2}-\beta_{∥}^{2}/\varepsilon_{⊥}\right)},$$

and, finally:

(A3) $$n_{\mathrm{eff}}^{p}=\sqrt{\varepsilon_{∥}+{\left(\beta_{∥}/\beta_{0}\right)}^{2}\left(1-\varepsilon_{⊥}/\varepsilon_{∥}\right)},$$

where $\beta_{∥}=\beta_{0}n_{\mathrm{SF}10}\sin\theta$ for beams incident from the glass prism.

To determine the interface-related effective index $n_{\mathrm{eff}}^{i}$, we considered an interface between MoS_2_ and some other material, which can be isotropic or uniaxial anisotropic with an axis normal to the interface. The axes were aligned so that the *x*-*y* plane was parallel to the interface, while the *x*-*z* plane was the plane of incidence. The positive direction of the *z* axis corresponded to incident and transmitted waves, with *z* = 0 corresponding to the interface. The boundary conditions for electromagnetic field include continuity of tangential component of the electric and magnetic field, as well as the continuity of the normal component of the electric displacement vector, which, in the case of p polarization, are written as:

(A4) $$\begin{array}{ccc} E_{1x}=E_{2x}, & H_{1y}=H_{2y}, & D_{1z}=D_{2z}. \end{array}$$

Next, we assumed $H_{1y}=\left[A_{1}\exp\left(i\beta_{1⊥}z\right)+B_{1}\exp\left(-i\beta_{1⊥}z\right)\right]\exp\left(i\beta_{∥}x-i\omega t\right)$ and $H_{2y}=\left[A_{2}\exp\left(i\beta_{2⊥}z\right)+B_{2}\exp\left(-i\beta_{2⊥}z\right)\right]\exp\left(i\beta_{∥}x-i\omega t\right)$. For simplification, we will drop the common factor $\exp\left(i\beta_{∥}x-i\omega t\right)$ hereafter. The boundary condition for *H_y_* reads *A*_1_ + *B*_1_ = *A*_2_ + *B*_2_. To fulfill the other boundary conditions, we determined electric field using the fourth Maxwell equation, which in CGS units reads:

(A5) curlH=−iωD.

The condition for *D_z_* is automatically fulfilled since $\partial_{x}H_{1y}=\partial_{x}H_{2y}$. Also, since $E_{x}=D_{x}/\varepsilon_{∥}=i\partial_{z}H_{y}/\left(\omega \varepsilon_{∥}\right)$, the boundary condition for *E_x_* is equivalent to:

(A6) $$\frac{\left(A_{1}-B_{1}\right)\beta_{1⊥}}{\varepsilon_{1∥}}=\frac{\left(A_{2}-B_{2}\right)\beta_{2⊥}}{\varepsilon_{2∥}}.$$

The last equation means that the same wave amplitudes *A*_1_, *B*_1_, *A*_2_, and *B*_2_ would satisfy the boundary conditions, with an effective isotropic layer replacing the actual anisotropic material as long as $\beta_{⊥}/\varepsilon_{∥}$ is conserved during the replacement. Using the dispersion equation for p-polarized waves (A1), we obtained:

(A7) $$\frac{\beta_{⊥}}{\varepsilon_{∥}}=\sqrt{\frac{\beta_{0}^{2}}{\varepsilon_{∥}}-\frac{\beta_{∥}^{2}}{\varepsilon_{∥}\varepsilon_{⊥}}},$$

Therefore, the dielectric function of the effective isotropic layer is determined as the solution of the equation:

(A8) $$\frac{\beta_{0}^{2}}{\varepsilon_{\mathrm{eff}}^{i}}-{\left(\frac{\beta_{∥}}{\varepsilon_{\mathrm{eff}}^{i}}\right)}^{2}=\frac{\beta_{0}^{2}}{\varepsilon_{∥}}-\frac{\beta_{∥}^{2}}{\varepsilon_{∥}\varepsilon_{⊥}}.$$
